# Prognostic factors for mesenchymal chondrosarcoma

**DOI:** 10.1051/sicotj/2024043

**Published:** 2024-11-08

**Authors:** Tomoya Masunaga, Shinji Tsukamoto, Kanya Honoki, Hiromasa Fujii, Akira Kido, Manabu Akahane, Yasuhito Tanaka, Andreas F. Mavrogenis, Costantino Errani, Akira Kawai

**Affiliations:** 1 Department of Orthopaedic Surgery, Nara Medical University 840, Shijo-cho Kashihara-city Nara 634-8521 Japan; 2 Department of Rehabilitation Medicine, Nara Medical University 840, Shijo-cho, Kashihara-city Kashihara-city Nara 634-8521 Japan; 3 Department of Health and Welfare Services, National Institute of Public Health 2-3-6 Minami Wako-shi Saitama 351-0197 Japan; 4 First Department of Orthopaedics, National and Kapodistrian University of Athens, School of Medicine 41 Ventouri Street 15562 Holargos Athens Greece; 5 Department of Orthopaedic Oncology, IRCCS Istituto Ortopedico Rizzoli Via Pupilli 1 40136 Bologna Italy; 6 Division of Musculoskeletal Oncology, National Cancer Center Hospital 5-1-1 Tsukiji Chuoku Tokyo 104-0045 Japan

**Keywords:** Mesenchymal chondrosarcoma, Adjuvant, Chemotherapy, Surgery, Radiotherapy

## Abstract

*Introduction*: Mesenchymal chondrosarcoma (MCS) is a malignant, biphasic, high-grade, primitive mesenchymal tumor that has a well-differentiated, organized hyaline component. MCS has a poor prognosis, and treatment recommended for localized MCS is based on wide resection while controversy remains regarding the efficacy of adjuvant chemotherapy and radiotherapy. In this study, we aimed to investigate the prognostic factors of MCS, especially the efficacy of adjuvant chemotherapy and radiotherapy for localized MCS. *Methods*: Eighty patients with MCS pathologically diagnosed between 2006 and 2022 from the Japanese National Bone and Soft Tissue Tumor Registry database were analyzed retrospectively. *Results*: Patients with distant metastases at presentation (*n* = 23) had significantly shorter survival than those without (*n* = 57) (5-year disease-specific survival 19.9% [95% confidence interval (CI): 5.6–50.7] vs. 79.8% [95% CI: 62.4–90.4]; *p* < 0.0001). In the group without distant metastasis at presentation (*n* = 57), R1 or R2 surgical margin was a risk factor for unfavorable local recurrence (hazard ratio (HR): 17.44 [95% CI: 2.17–139.98]; *p* = 0.007). There was no correlation between adjuvant radiotherapy and local recurrence rate (HR 5.18 [95% CI: 0.99–27.12]; *p* = 0.051). R1 or R2 surgical margin was a risk factor for unfavorable disease-specific survival (HR 17.42 [95% CI: 2.18–138.90]; *p* = 0.007). There was no correlation between adjuvant chemotherapy and disease-specific survival (HR 0.99 [95% CI: 0.28–3.47]; *p* = 0.990). *Discussion*: Patients with MCS and distant metastases at presentation have a poor prognosis, and wide resection is important for the treatment of localized MCS. The efficacy of adjuvant radiotherapy and chemotherapy could not be determined because of the small number of patients.

## Introduction

Mesenchymal chondrosarcoma (MCS) is a malignant, high-grade, biphasic, primitive mesenchymal tumor containing an organized, well-differentiated hyaline component [1]. MCS makes up 2–4% of all chondrosarcomas [1] and likely originates from immature chondroblasts, which differentiate into well-differentiated cartilage, frequently including enchondral ossification [1]. In almost all MCS cases a recurrent, highly specific gene fusion occurs between HEY1 and NCOA2 [2]. MCS generally arises in young adults in their 20s and 30s [3], with a slight preponderance of males [4–6], developing in soft tissue, bone, or intracranial sites [4–6]. The most common bone sites are the craniofacial region, ribs, spine, ilium, and femur [4–6]. Around one-third of MCSs arise in extraosseous soft tissues such as the central nervous system, often the meninges [4–6]. The most frequent soft tissue sites are the head and neck, then the lower extremities [4–6]. The 5-year survival rate is approximately 50–70% [4–6], and 15–25% of cases have metastatic disease at diagnosis [4, 5].

Although wide resection is the basic treatment for localized MCS, the efficacy of adjuvant chemotherapy is controversial [4, 5]. The National Comprehensive Cancer Network guidelines recommend that treatment of localized MCS should follow the Ewing sarcoma protocol (adjuvant/neoadjuvant chemotherapy plus wide resection) [7]. The European Society for Medical Oncology guidelines states that “Adjuvant/neoadjuvant chemotherapy combining anthracycline and alkylating agents can also be considered for localized MCS” [8]. However, because of its rarity, there have only been retrospective studies, and the effect of (neo-)adjuvant chemotherapy on localized MCS has not been examined in randomized controlled trials (RCTs).

Adjuvant radiotherapy is recommended to achieve good local control of MCS even when clear surgical margins are not secured [4, 9]. However, like (neo-)adjuvant chemotherapy, only retrospective studies have been performed, and no RCTs on the effect of adjuvant radiotherapy on localized MCS, so its effect is unclear.

We investigated the prognostic factors of MCS and the efficacy of adjuvant chemotherapy and adjuvant radiotherapy for localized MCS.

## Methods

Ninety-two patients with MCS pathologically diagnosed between January 2006 and December 2022 were selected from the Japanese National Bone and Soft Tissue Tumor Registry database [10, 11]. After excluding 12 patients with inadequate records, the 80 remaining patients were analyzed retrospectively. Data collected were: age, sex, date of diagnosis, previous surgery, tissue of origin, tumor site, tumor size, distant metastases at diagnosis, biopsy method, surgical margins [microscopically negative margins are defined as R0; macroscopically negative, but microscopically positive margins as R1; and macroscopically positive margins as R2 [12], adjuvant radiotherapy, adjuvant chemotherapy, distant metastases, local recurrence, disease-specific survival, and follow-up period. According to a recent systematic review, carbon ion radiotherapy (CIRT) improves local control and survival rates for chondrosarcoma compared with conventional and proton therapy [13]. Therefore, local treatment was classified into four categories: R0 surgical margin, R1 or R2, CIRT, and no surgery.

Local recurrence-free survival was defined as the interval between surgery and local recurrence or final follow-up, while distant metastasis-free survival was defined as the interval between diagnosis and distant metastasis or final follow-up. Disease-specific survival was defined as the interval between diagnosis and death from disease or final follow-up. To examine the correlation between each variable and survival, univariate analysis was performed using Cox proportional hazards regression analysis and Kaplan–Meier survival analysis (a log-rank test). Statistical significance was set at *p* < 0.05. Analyses were performed using JMP 17 (SAS Institute Inc., Cary, NC, USA).

## Results

The study group comprised 43 (53.8%) males and 37 (46.3%) females with a median age of 34 years (interquartile range [IQR], 26.8–64.8). Sixteen (20%) patients previously underwent surgery. Forty-four cases (55%) were of skeletal origin and 36 (45%) were of extra-skeletal origin. Tumor location was head and neck in eight patients (two skulls, one maxilla, two necks, and three faces), trunk in 46 (eight ribs, six scapulae, two cervical spines, three thoracic spine, three lumbar spines, five sacra, three ilia, one ischium, one pubis, two thoracic/mediastinal, one shoulder, one chest wall, one back, one abdominal wall, four retroperitoneum, one lumbar, one inguinal, and two buttocks), and limbs in 26 (two humeri, three femur, three tibias, one fibula, one upper arm, one forearm, 10 thighs, and five lower legs). Among the 80 patients, 57 (71.3%) had no distant metastases at diagnosis, and the remaining 23 (28.8%) had distant metastases at diagnosis. Median tumor size was 7 cm (IQR, 5–10). Disease-specific survival was 94.3% at 1 year, 81.4% at 2 years, and 62.7% at 5 years. Median disease-specific survival was 31.5 months (IQR, 14–63.8).

### Patients without metastases at diagnosis

Fifty-seven patients had no distant metastases at diagnosis. The median age was 36 years (IQR, 27.5–65.5), and nine patients (15.8%) previously underwent surgery. There were 31 patients (54.4%) with skeletal origin and 26 (45.6%) with extra-skeletal origin. Tumor location was head and neck in six patients (one skull, one maxilla, two cervical, and two facial), trunk in 33 (six ribs, five scapulae, two cervicals, three thoracics, two lumbar, four sacral, one ischium, one shoulder, one chest wall, one back, one abdominal wall, two retroperitoneum, one lumbar, one groin, and two buttocks), and limbs in 18 (two femora, three tibia, one fibula, one upper arm, one forearm, seven thigh, and three lower leg). The median tumor size was 6 cm (IQR, 4.4–9). Nine patients (15.8%) were diagnosed by needle biopsy, 43 (75.4%) by incisional biopsy, and five (8.8%) without biopsy.

Regarding surgery, 38 underwent R0 resection, two underwent R1 resection, two underwent R2 resection, and seven underwent CIRT. Eight did not undergo surgery, and three of these received proton radiotherapy. Four patients received adjuvant radiotherapy: all four received postoperative radiotherapy, and none received preoperative radiotherapy. Twenty-four patients (42.1%) received adjuvant chemotherapy, 15 received AI, seven received VDC-IE, and two received methotrexate-adriamycin-cisplatin.

For the analysis of local recurrence, eight patients who did not undergo surgery or CIRT were excluded, leaving 49 patients included. Seven patients experienced local recurrence (14.3%), and the median surgery-to-local recurrence period was 32 months (IQR, 7–44). Distant metastases occurred in 22 patients (38.6%), and the median time from diagnosis to distant metastasis was 22 months (IQR, 9.8–30.3). Ten patients (17.5%) died of tumors, and the median time from diagnosis to disease-related death was 39 months (IQR, 19.5–81.8). The median disease-specific survival and follow-up periods were both 33 months (IQR, 16–74).

Regarding local recurrence, in univariate analysis, R1 or R2 surgical margin was a risk factor for unfavorable local recurrence (HR 17.44 [95% CI: 2.17–139.98]; *p* = 0.007) (Table 1). There was no correlation between adjuvant radiotherapy and local recurrence rate (HR 5.18 [95% CI: 0.99–27.12]; *p* = 0.051) (Table 1). Of the four patients given adjuvant radiotherapy, two (50.0%) had local recurrence, and of the 45 patients without adjuvant radiotherapy, five (11.1%) had local recurrence. There was no correlation between adjuvant chemotherapy and local recurrence rate (HR 1.57 [95% CI: 0.35–7.01]; *p* = 0.557) (Table 1). Regarding distant metastasis, in univariate analysis large size was a risk factor for unfavorable distant metastasis (HR 1.14 [95% CI: 1.04–1.26]; *p* = 0.007) (Table 2). There was no correlation between adjuvant chemotherapy and distant metastasis rate (HR 1.76 [95% CI: 0.75–4.09]; *p* = 0.192) (Table 2). Among 24 patients who received adjuvant chemotherapy, 12 (50.0%) had distant metastasis, and among 33 patients who did not, 10 (30.3%) had distant metastasis. Regarding disease-specific survival, in univariate analysis, R1 or R2 surgical margin was a risk factor for unfavorable disease-specific survival (HR 17.42 [95% CI: 2.18–138.90]; *p* = 0.007) (Table 3). No correlation was found between adjuvant chemotherapy and disease-specific survival (HR 0.99 [95% CI: 0.28–3.47]; *p* = 0.990) (Table 3). Of the 24 patients who received adjuvant chemotherapy, 11 (45.8%) remained disease-free, seven (29.2%) were alive with disease, five (20.8%) died of the tumor, and one (4.2%) died of another disease. Of the 33 patients who did not receive adjuvant chemotherapy, 21 (63.6%) remained disease-free, seven (21.2%) were alive with disease, five (15.2%) died of tumor, and no patients died of another disease.

**Table 1:** Univariate Cox regression analysis of local recurrence-free survival in patients who had localized mesenchymal chondrosarcoma and underwent surgery or CIRT.

Variable (*n* = 49)	No. of patients	Hazard ratio (95% CI)	*p*-value
Age (years)	49	1.01 (0.97–1.05)	0.626
Sex			
Male	26	1	
Female	23	0.36 (0.07–1.86)	0.223
Year of diagnosis			
2006 to 2013	17	1	
2014 to 2022	32	6.39 (0.75–54.75)	0.091
Previous surgery			
No	42	1	
Yes	7	3.49 (0.78–15.66)	0.103
Tissue of origin			
Skeletal	26	1	
Extra-skeletal	23	1.60 (0.36–7.16)	0.539
Site			
Head and neck	5	1	
Trunk	27	0.35 (0.06–1.94)	0.230
Limbs	17	0.15 (0.01–1.63)	0.118
Tumor size (cm)	49	0.98 (0.79–1.22)	0.861
Surgical margin			
R0	38	1	
R1 or R2	4	17.44 (2.17–139.98)	0.007*
CIRT	7	1.96 (0.21–17.89)	0.550
Adjuvant radiotherapy			
No	45	1	
Yes	4	5.18 (0.99–27.12)	0.051
Adjuvant chemotherapy			
No	27	1	
Yes	22	1.57 (0.35–7.01)	0.557

*Statistically significant. CI, confidence interval; CIRT, carbon ion radiotherapy.

**Table 2:** Univariate Cox regression analysis of distant metastasis-free survival in patients with localized mesenchymal chondrosarcoma

Variable (*n* = 57)	No. of patients	Hazard ratio (95% CI)	*p*-value
Age (years)	57	1.00 (0.98–1.02)	0.861
Sex			
Male	30	1	
Female	27	0.45 (0.19–1.06)	0.067
Year of diagnosis			
2006 to 2013	19	1	
2014 to 2022	38	0.84 (0.36–1.96)	0.692
Previous surgery			
No	48	1	
Yes	9	1.67 (0.61–4.53)	0.317
Tissue of origin			
Skeletal	31	1	
Extra-skeletal	26	0.63 (0.26–1.51)	0.299
Site			
Head and neck	6	1	
Trunk	33	1.40 (0.31–6.28)	0.663
Limbs	18	2.03 (0.43–9.63)	0.374
Tumor size (cm)	57	1.14 (1.04–1.26)	0.007*
Surgical margin			
R0	38	1	
R1 or R2	4	1.47 (0.34–6.46)	0.609
CIRT	7	0.77 (0.23–2.64)	0.680
No surgery	8	0	0.999
Adjuvant radiotherapy			
No	53	1	
Yes	4	0.71 (0.10–5.35)	0.741
Adjuvant chemotherapy			
No	33	1	
Yes	24	1.76 (0.75–4.09)	0.192

*Statistically significant. CI, confidence interval; CIRT, carbon ion radiotherapy.

**Table 3:** Univariate Cox regression analysis of disease-specific survival in patients with localized mesenchymal chondrosarcoma.

Variable (*n* = 57)	No. of patients	Hazard ratio (95% CI)	*p*-value
Age (years)	57	1.03 (1.00–1.06)	0.085
Sex			
Male	30	1	
Female	27	0.35 (0.09–1.34)	0.125
Year of diagnosis			
2006 to 2013	19	1	
2014 to 2022	38	2.41 (0.51–11.50)	0.269
Previous surgery			
No	48	1	
Yes	9	1.82 (0.36–9.13)	0.465
Tissue of origin			
Skeletal	31	1	
Extra-skeletal	26	0.80 (0.22–2.85)	0.726
Site			
Head and neck	6	1	
Trunk	33	0.45 (0.09–2.37)	0.345
Limbs	18	0.11 (0.01–1.33)	0.083
Tumor size (cm)	57	1.04 (0.88–1.23)	0.647
Surgical margin			
R0	38	1	
R1 or R2	4	17.42 (2.18–138.90)	0.007*
CIRT	7	3.14 (0.56–17.71)	0.196
No surgery	8	1.74 (0.20–14.95)	0.615
Adjuvant radiotherapy			
No	53	1	
Yes	4	4.65 (0.48–45.16)	0.186
Adjuvant chemotherapy			
No	33	1	
Yes	24	0.99 (0.28–3.47)	0.990

*Statistically significant. CI, confidence interval; CIRT, carbon ion radiotherapy.

### Patients with metastases at diagnosis

Twenty-three patients had distant metastases at diagnosis. Seventeen patients had lung metastases, eight had bone metastases, one had liver metastases, two had adrenal metastases, and two had pancreatic metastases. Thirteen (56.5%) were male and 10 (43.5%) were female, median age was 32 years (IQR, 25–57), and seven (30.4%) had previous surgery. There were 13 tumors (56.5%) of skeletal origin and 10 (43.5%) of extra-skeletal origin. Tumor location was head and neck in two patients (one skull and one facial), trunk in 13 (two ribs, one scapula, one lumbar spine, one sacrum, three ilia, one pubis, two thoracic/mediastinum, and two retroperitoneum), and limbs in eight (two humerus, one femur, three thigh, and two lower leg). The median tumor size was 10 cm (IQR, 7–13). Three (13.0%) patients were diagnosed by needle biopsy, 14 (60.9%) by incisional biopsy, and six (26.1%) without biopsy.

Regarding surgery for the primary tumor, six patients underwent R0 resection and one patient underwent R1 resection. Two patients underwent CIRT and 14 did not undergo surgery. Postoperative adjuvant radiotherapy was administered to two patients. Two patients received palliative radiotherapy for the primary tumor without surgery. Twenty patients (87.0%) received chemotherapy for advanced disease: six received vincristine-adriamycin-cyclophosphamide (VDC), 11 received ifosfamide-etoposide (IE), two received trabectedin, one received vincristine-actinomycin D-cyclophosphamide, six received adriamycin-ifosfamide (AI), three received gemcitabine-docetaxel, three received adriamycin-cisplatin, three received adriamycin, three received eribulin, and four received pazopanib. Five patients underwent metastasectomy, including three patients who underwent pneumonectomy and two patients who underwent bone resection.

**Figure 1:**
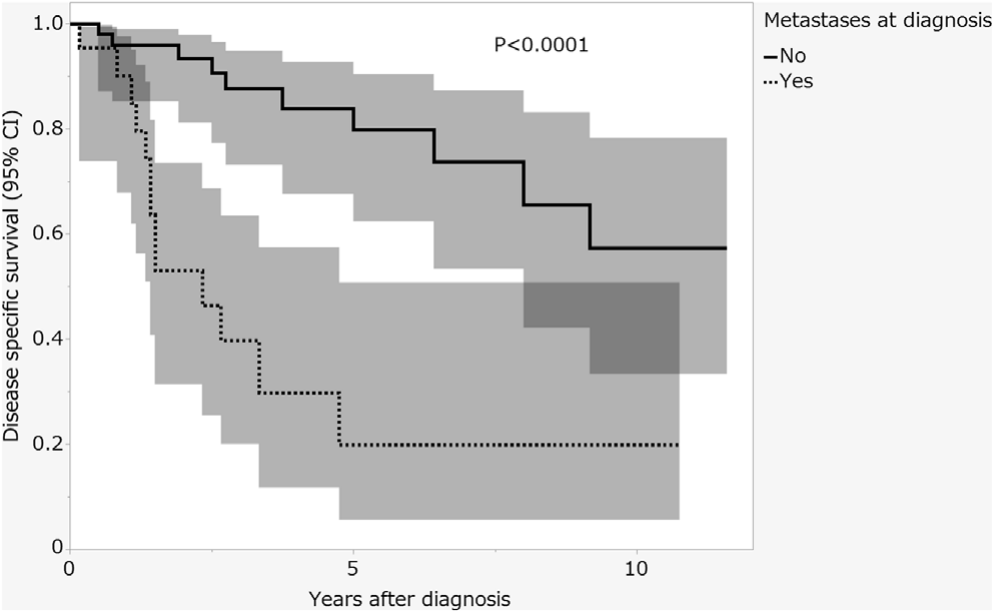
Disease-specific survival rates of patients with and without distant metastases at diagnosis. The shading around the curves represents the 95% confidence interval.

Disease-specific survival was 90.2% at 1 year, 53.0% at 2 years, and 19.9% at 5 years. Median disease-specific survival was 18 months (IQR, 10–34). Univariate analysis showed significantly shorter survival in the group with distant metastases at presentation compared with the group without (5-year disease-specific survival 19.9% [95% CI: 5.6–50.7] vs. 79.8% [95% CI: 62.4–90.4]; *p* < 0.0001; Figure 1).

## Discussion

Consistent with previous reports, survival was significantly shorter in patients presenting with distant metastases [5, 14, 15]. In patients with localized MCS, the risk factor for local recurrence was inadequate surgical margins, and the local control effect of adjuvant radiotherapy was limited. The risk factor for distant metastasis was a large size, and the poor prognostic factor for survival was inadequate surgical margins. Adjuvant chemotherapy had limited effects in preventing distant metastasis or improving survival.

Disease-specific survival was 63% at 5 years in 80 MCS patients treated from 2006 to 2022 in the National Bone and Soft Tissue Tumor Registry database. A systematic literature review from 1994 to 2014 by Xu et al. showed a 5-year survival rate of 55% among 107 MCS patients [4], while a European Musculo-Skeletal Oncology Society (EMSOS) study of 107 MCS patients treated from 1971 to 2012 showed a 5-year survival of 70% [5]. In 205 MCS patients from the Surveillance, Epidemiology, and End Results (SEER) data from 1973 to 2011, overall survival was 51% at 5 years [6].

Our univariate analysis showed that large size was a risk factor for distant metastasis. In a multivariate analysis of 205 MCS patients from the SEER dataset, a 1 cm increase in tumor size was an independent predictor of death from disease (HR, 1.16 [95% CI, 1.09–1.23]; *p* = 0.001) [6].

Univariate analysis showed that R1 or R2 surgical margin was a risk factor for local recurrence and reduced disease-specific survival. Nakashima et al. reported that 33/41 patients (80.5%) who underwent intralesional resection experienced local recurrence, whereas only nine of 22 patients (40.9%) who underwent wide resection did so [16]. The EMSOS study of 95 patients with localized MCS reported higher local recurrence in patients after R1 resection compared with others who underwent R0 resection (27% vs. 2%; *p* = 0.002) [5]. Meanwhile, in the review by Xu et al., R0 resection significantly improved both overall and local recurrence-free survival in 107 MCS patients [4]. Tsuda et al. reported that in 40 patients with localized MCS, positive margins were significantly associated with increased local recurrence [15]. Furthermore, in 26 MCS patients, 10-year survival was significantly increased in patients with R0 resection compared with those without (27% vs. 0%; *p* = 0.0007) [14]. In 19 localized MCS patients, tumor resection was associated with significantly prolonged survival (183 vs. 17 months; *p* = 0.003) [17]. Thus, for patients with localized MCS, tumor resection with adequate margins is important to reduce local recurrence and prolong survival.

Univariate analysis in this study showed no correlation between adjuvant radiotherapy and local recurrence rate. However, postoperative adjuvant radiotherapy reportedly improves local recurrence-free survival in patients with positive surgical margins [4, 9]. Kawaguchi et al. analyzed 28 patients with localized MCS, 10 of whom received radiotherapy, and found a significant difference in local recurrence-free survival between those who received radiotherapy and those who did not (*p* = 0.037), with radiotherapy associated with improved local recurrence-free survival [9]. The review by Xu et al. found that adjuvant radiotherapy reduced local recurrence in patients with positive surgical margins (*p* = 0.013) [4].

Univariate analysis showed no correlation between adjuvant chemotherapy and distant metastasis rate or disease-specific survival. Adjuvant chemotherapy may reduce the risk of local recurrence and distant metastasis in localized MCS [4, 5, 14]. However, data on the effect of chemotherapy on survival remain inconsistent. Some studies report a reduced risk of death [5], while others report no association [4, 9, 14, 15].

Cesari et al. studied 21 MCS patients who achieved complete surgical remission, and reported 76% 10-year disease-free survival in patients treated with chemotherapy (*n* = 9) and 17% in those without (*n* = 12) (*p* = 0.008) [14]; however, there was no significant difference in 10-year overall survival between the groups (31% vs. 19%) [14]. Similarly, Xu et al. found that adjuvant chemotherapy improved event-free survival (*p* = 0.046), but did not improve overall survival (*p* = 0.139) [4].

Nonetheless, in the EMSOS study of 95 patients with localized MCS, those who received chemotherapy [52/54 patients (96%) received an anthracycline, 38/54 (70%) also received alkylating agents] had a reduced risk of recurrence (HR 0.482 [95% CI: 0.213–0.996]; *p* = 0.046) and death (HR 0.445 [95% CI: 0.256–0.774]; *p* = 0.004) [5]. They recommended (neo-)adjuvant chemotherapy with doxorubicin and ifosfamide or cisplatin for localized MCS [5].

In previous studies, four MCS patients received high-dose methotrexate-based preoperative chemotherapy, but none responded [18]; two MCS patients received preoperative chemotherapy, but neither responded [19]. However, among seven MCS patients given preoperative chemotherapy, >50% tumor volume regression or histologic devitalization of >50% occurred in four patients [3]. Tsuda et al. reported that three of four MCS patients administered preoperative chemotherapy showed stable disease and one showed a partial response [15]. Thus, the efficacy of preoperative chemotherapy remains uncertain.

A sub-analysis of a phase two study of the efficacy of trabectedin in advanced-stage MCS treated three patients with a median progression-free survival of 12.5 months [20]. In addition, a prospective phase II clinical study (NCT04305548) by the Italian Sarcoma Group is currently underway to investigate the activity of trabectedin in patients with advanced-stage MCS.

This study has several limitations. First, because of its retrospective nature, there were indication biases for adjuvant radiotherapy and chemotherapy. The random allocation of RCTs avoids many of these biases; however, RCTs are extremely difficult for rare cancers like MCS, thus this study aimed to investigate prognostic factors. Second, the small number of patients and events did not allow for multivariate analysis. The results should be interpreted with caution because of the lack of adjustment for confounding factors. Additionally, the small number of patients may cause the possibility of type 2 errors, and significant factors may emerge with increasing numbers of patients. In the future, an international collaborative study should be conducted to increase the number of patients and re-evaluate the results of the present study.

## Conclusion

MCS patients with distant metastases at presentation had shorter survival than patients with localized MCS; in localized MCS, the risk factor for local recurrence was inadequate surgical margins, the risk factor for distant metastases was large size, and the poor prognostic factor for survival was inadequate surgical margins. Therefore, wide resection is important when treating localized MCS. The effects of adjuvant chemotherapy and radiotherapy were inconclusive because of the small number of patients.

## Data Availability

The datasets generated and/or analyzed during the current study are not publicly available due to privacy considerations but are available from the corresponding author upon reasonable request.
